# The dynamic architecture of the metabolic switch in *Streptomyces coelicolor*

**DOI:** 10.1186/1471-2164-11-10

**Published:** 2010-01-06

**Authors:** Kay Nieselt, Florian Battke, Alexander Herbig, Per Bruheim, Alexander Wentzel, Øyvind M Jakobsen, Håvard Sletta, Mohammad T Alam, Maria E Merlo, Jonathan Moore, Walid AM Omara, Edward R Morrissey, Miguel A Juarez-Hermosillo, Antonio Rodríguez-García, Merle Nentwich, Louise Thomas, Mudassar Iqbal, Roxane Legaie, William H Gaze, Gregory L Challis, Ritsert C Jansen, Lubbert Dijkhuizen, David A Rand, David L Wild, Michael Bonin, Jens Reuther, Wolfgang Wohlleben, Margaret CM Smith, Nigel J Burroughs, Juan F Martín, David A Hodgson, Eriko Takano, Rainer Breitling, Trond E Ellingsen, Elizabeth MH Wellington

**Affiliations:** 1 Center for Bioinformatics Tübingen, Department of Information and Cognitive Sciences, University of Tübingen, Sand 14, D-72076 Tübingen, Germany; 2 Department of Biotechnology, Norwegian University of Science and Technology (NTNU), Sem Sælandsvei 6-8, N-7491 Trondheim, Norway; 3 Department of Biotechnology, SINTEF Materials and Chemistry, Sem Sælandsvei 2a, N-7465 Trondheim, Norway; 4 Groningen Bioinformatics Centre, University of Groningen, Kerklaan 30, 9751 NN Haren, the Netherlands; 5 Department of Microbial Physiology, University of Groningen, Kerklaan 30, 9751 NN Haren, the Netherlands; 6 Department of Chemistry, University of Warwick, Gibbet Hill Road, Coventry CV4 7AL, UK; 7 Instituto de Biotecnología de León, INBIOTEC, Parque Científico de León, Av. Real 1, 24006 León, Spain, and Área de Microbiología, Universidad de León, Spain; 8 Department of Microbiology/Biotechnology, University of Tübingen, Auf der Morgenstelle 28, D-72076 Tübingen, Germany; 9 School of Medical Sciences, Institute of Medical Sciences, University of Aberdeen, Foresterhill, Aberdeen AB25 2ZD, UK; 10 Department of Biological Sciences, University of Warwick, Gibbet Hill Road, Coventry CV4 7AL, UK; 11 Warwick Systems Biology Centre, University of Warwick, Coventry House, Coventry, CV4 7AL, UK; 12 Microarray Facility Tübingen, Calwer Strasse 7, D-72076 Tübingen, Germany

## Abstract

**Background:**

During the lifetime of a fermenter culture, the soil bacterium *S. coelicolor *undergoes a major metabolic switch from exponential growth to antibiotic production. We have studied gene expression patterns during this switch, using a specifically designed Affymetrix genechip and a high-resolution time-series of fermenter-grown samples.

**Results:**

Surprisingly, we find that the metabolic switch actually consists of multiple finely orchestrated switching events. Strongly coherent clusters of genes show drastic changes in gene expression already many hours before the classically defined transition phase where the switch from primary to secondary metabolism was expected. The main switch in gene expression takes only 2 hours, and changes in antibiotic biosynthesis genes are delayed relative to the metabolic rearrangements. Furthermore, global variation in morphogenesis genes indicates an involvement of cell differentiation pathways in the decision phase leading up to the commitment to antibiotic biosynthesis.

**Conclusions:**

Our study provides the first detailed insights into the complex sequence of early regulatory events during and preceding the major metabolic switch in *S. coelicolor*, which will form the starting point for future attempts at engineering antibiotic production in a biotechnological setting.

## Background

The switch from primary metabolism (exponential growth) to secondary metabolism (stationary growth) upon nutrient starvation is commonly found in most microorganisms [1]. The phenomenon has been known for a long time, but new details of function and regulation of the "metabolic switch" continue to emerge as we begin to apply postgenomic technology to the analysis. Understanding the switch to secondary metabolism is of major importance in biotechnology, where it can contribute to the optimized production of commercially relevant secondary metabolites, such as antibiotics.

Here we have used the soil bacterium *Streptomyces coelicolor*, the model organism of the antibiotics producing genus *Streptomyces*, to dissect its metabolic switch in unprecedented detail. Reproducible growth of the filamentous *Streptomyces *species has been a challenge, and especially producing the same quantity of antibiotics in each fermentation has been a major hurdle for conducting systems biology experiments in these species. Some short gene expression time series have been reported [2-5], the largest being a study by Lian et al. [5], with 13 time points at 1- to 3-hour intervals from 15 hours to 42 hours comparing the *S. coelicolor *M145 wild type to a pleiotropic regulator mutant, but these experiments were conducted in shaker flasks, where cultivation conditions, such as pH and dissolved oxygen, were not held constant, resulting in irreproducible fluctuations in growth from run to run.

**Figure 1:**
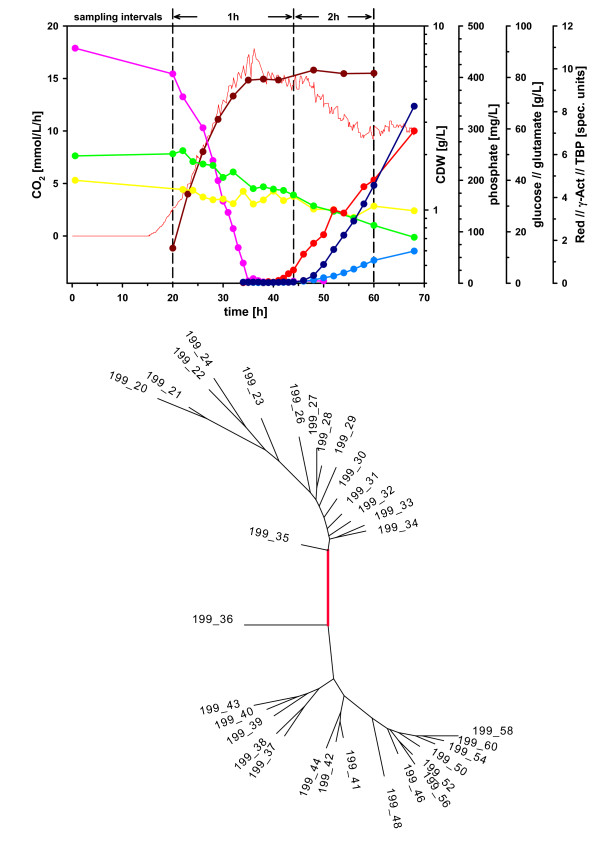
**Characterization of the fermentation time series samples**. A. Major biochemical parameters of the fermentation run. The figure shows the increase in biomass (brown), phosphate depletion (pink), the decrease of glucose (yellow) and glutamate (green) levels, as well as the production of the antibiotics undecylprodigiosin (red), actinorhodin (light blue), and total blue pigment (dark blue). Continuous measurements of CO_2 _production are indicated by the thin red line. The sampling intervals for the gene expression measurements are indicated at the top. B. Hierarchical clustering of the 32 samples along the time course. Each leaf of the tree corresponds to one sample and the labels indicate the time (in hours) after inoculation at which the sample was harvested. The clustering of the 32 samples is based on the expression profiles of 322 transcripts with highest regularized variance. A neighbor joining tree was produced from the pairwise Euclidean distances between the expression profiles of all samples.

We have overcome these problems by using a glutamate-based minimum medium which is phosphate limited and by strictly controlling the growth conditions for the fermentation of *S. coelicolor*. Cells were grown up to 68 hours after inoculation and monitored for cell dry weight, CO_2_, phosphate and the accumulation of two antibiotics, actinorhodin and undecylprodigiosin (Figure 1A). From 20 hours, samples were taken every hour up to 44 hours, and every two hours afterwards until 60 hours. Global gene expression profiles were acquired for each sample using newly designed customized Affymetrix genechips. This is the first time that the necessary reproducible fermentation has been achieved for any filamentous bacterium. The detailed transcriptome time series, which is deepest resolved transcriptomic data ever produced for an actinomycete, is currently complemented by metabolome and proteome analysis. It forms the starting point for a systems biology approach for this class of organisms which is of greatest biotechnological relevance.

## Results

Unsupervised clustering analysis revealed a strong structure in the data: on the one hand, when clustering the samples, adjacent time points cluster closely together (Figure 1B), and the strongest change in gene expression occurs between time points 35 and 36 hours; here the two samples are clearly separated on the clustering tree despite being just 1 hour apart along the growth curve. This interval coincides with the moment when phosphate in the medium is depleted and corresponds to the classical metabolic switch. On the other hand, when clustering the gene expression profiles, several major groups of genes emerge as showing highly correlated gene expression patterns. A more detailed analysis of this correlation structure identified a very fine-grained dynamic pattern of gene expression. In addition to two large groups of genes that showed consistent gradual increase or decrease of expression levels, respectively, we observed several clusters that showed a more complex, transient upregulation. Detailed analysis of each of these clusters revealed that they consist of biologically coherent groups of genes, often dominated by a few large operons, and reveal an unexpectedly complex series of switching events well before and after the classical metabolic switch.

The expression measurements were validated by qRT-PCR using six genes that showed highly characteristic and very different profiles. The resulting time course profiles are highly correlated to those obtained by the microarray experiments (Pearson correlation between 0.74 and 0.93; see Additional File 1). To verify reproducibility, the fermentation was repeated for a total of four biological replicates and microarray data collected for 8 time points in each of the fermentations. An evaluation of the variance between the biological replicates revealed an average standard deviation across all replicates of less than 6% for biomass concentration and remaining phosphate levels in the growth medium, and less than 10% for the production levels of undecylprodigiosin and actinorhodin. Similar correlations were seen for groups of differentially expressed genes, with between-fermentor correlations for individual clusters of genes ranging from 0.48 to 0.94 (average 0.72), indicating excellent synchronicity between replicates.

**Figure 2:**
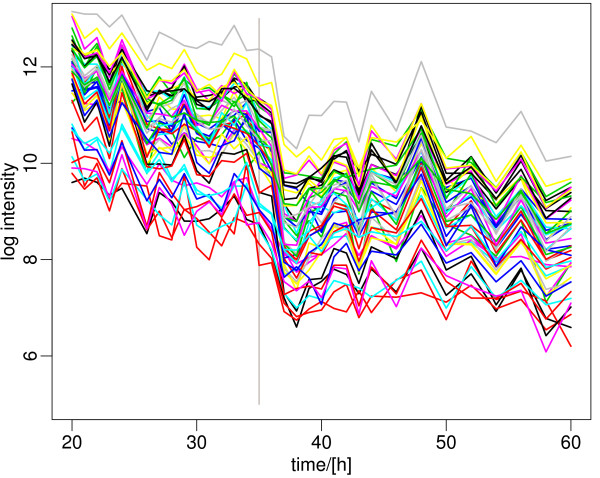
**Representative genes of the ribosomal gene cluster, showing continuous decrease in expression**. The major expression change happens after 35 h, when phosphate is depleted in the medium (grey vertical line). A list of the genes and their expression data is included in Additional File 2.

As expected, the strongest continually decreasing cluster of genes is dominated by ribosomal proteins and other proteins with functions in protein biosynthesis. These "house-keeping" genes are highly expressed initially and then gradually decline as the cells enter transition and stationary phase (Figure 2). This closely mimics the pattern observed in the stringent response [6], and also shows strong similarity with the patterns seen in the diauxic shift of baker's yeast [7].

**Figure 3:**
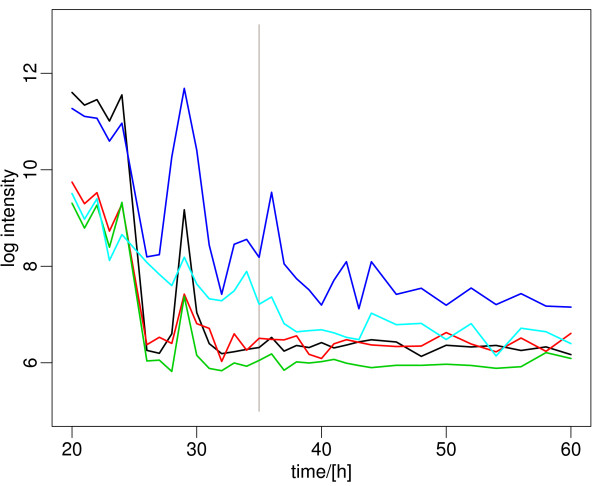
**Representative genes of the nitrogen metabolism gene cluster**. A clear switch is visible already at 24 h, and after 31 h the genes are approaching background expression levels. A list of the genes and their expression data is included in Additional File 2.

The earliest gene expression change along the growth curve was evidenced by a cluster of genes that are mainly involved in nitrogen metabolism, including glutamine synthetase I and II, and the signaling protein glnK (Figure 3; [8]): it starts off at high expression at the earliest time points (20 h after inoculation = very early exponential phase). Expression of this cluster rapidly declines at 24 h, shows another rapid transient peak around 28 h, and then levels off at very low expression. This expression pattern of the nitrogen metabolism genes is unexpected since it differs drastically from protein data and activity measurements made in flask cultures or on solid media [9], as well as earlier expression profiles in a different medium [5]. The early expression of these genes is particularly surprising as the medium contains excess glutamate and is not limited in nitrogen. The nitrogen metabolism genes are regulated by *glnR *[10], which shows the same expression trend (see below).

**Figure 4:**
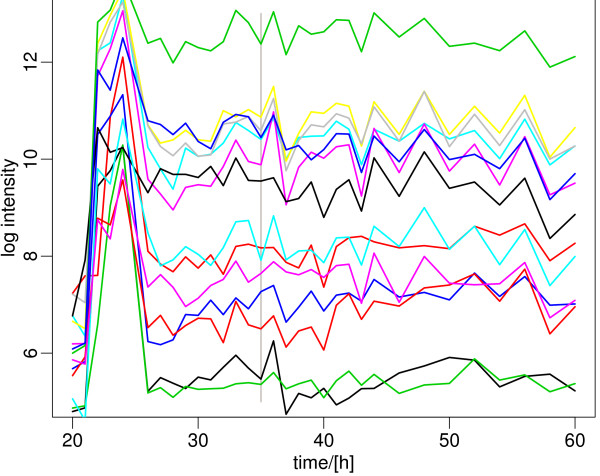
**Representative genes of the CPK antibiotics gene cluster**. A strong transient expression peak is seen around 24 h, for all of the genes, and many of them remain at constantly elevated expression levels afterwards. A list of the genes and their expression data is included in Additional File 2.

Overlapping with the gradual expression change in nitrogen metabolism, we observe a first and very striking switch in secondary metabolism: the CPK antibiotic biosynthesis genes (SCO6268-SCO6285 (except SCO6269); [11]) rapidly increase in expression at 22 h, and then equally rapidly decline again at 25 h, albeit not returning to their pre-25 h levels in all cases (Figure 4). As can be seen in the figure, this transient peak in gene expression of antibiotics biosynthesis genes (encoding the core type I PKS domains and several modifying genes) is strikingly preceded by a slightly earlier peak in expression of the main transcriptional activator of the cluster, SCO6269 (*cpkO*). Interestingly, the direct neighbors of the CPK genes (SCO6265-6267), which are the γ-butyrolactone receptor *scbR *and the γ-butyrolactone synthesis genes *scbA *and *scbB *[12], show a similar pattern, with a rapid increase by 21 h, a peak in expression at 22 h, followed by a sharp decline after one hour. The butyrolactone system has been shown earlier to regulate *cpkO *via ScbR [13]. This highlights our ability of identifying putative regulatory connections by examining densely sampled time courses. A similar highly informative shift in gene expression is observed for *scoT *(SCO6287), which is the enzyme thought to cleave off the expanding polyketide core carbon chains at the end of the biosynthetic pathway [14]. *scoT *expression increases at 23 h, one hour later than the core biosynthesis genes (not shown).

**Figure 5:**
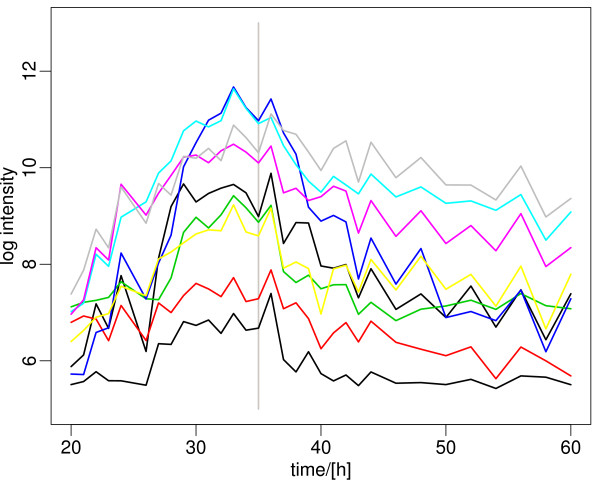
**Representative genes of the cluster of development related genes**. These genes, which are involved in morphogenesis and the sporulation process, show a continuous increase in expression early in the time course, but are repressed immediately before the major metabolic switch happens. A list of the genes and their expression data is included in Additional File 2.

One additional cluster shows a highly interesting switching pattern preceding the traditional metabolic switch: expression of these genes gradually increases to a peak around 33 h, and subsequently decreases again to the original level (Figure 5). The genes involved are a more heterogeneous group than in any of the other clusters; they include the nitrate reductase cluster (SCO0212-0220, only excluding SCO0214/15) and many developmental genes, including the chaplins [15], *bldN *[16] and *whiH *[17]. This is the first observation of these developmental genes expressed during a metabolic switch and is particularly significant as *S. coelicolor *does not produce spores in liquid media. The expression of these genes increases as though the cells prepare for differentiation, but some signal stops their expression around 33 h so that differentiation does not occur. It would be of extreme interest to analyze the expression of this group of developmental genes in other *Streptomyces *species, for example *Streptomyces griseus*, which do sporulate in liquid culture [18].

**Figure 6:**
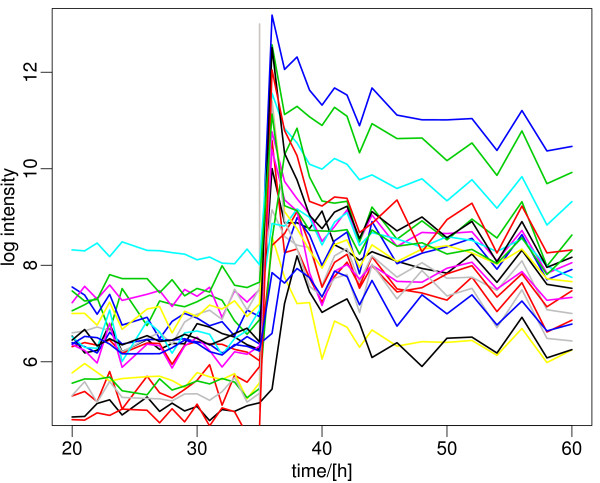
**Genes upregulated by phosphate depletion**. These include the regulatory genes *phoP, phoU *and *phoR*, genes of the phosphate transport cluster (*pstABCS*), as well as 13 other genes identified as activated by PhoP in the *pho *regulon [20-22]. The dramatic change in expression of these genes coincides exactly with the major metabolic switch induced by phosphate depletion. A list of the genes and their expression data is included in Additional File 2.

**Figure 7:**
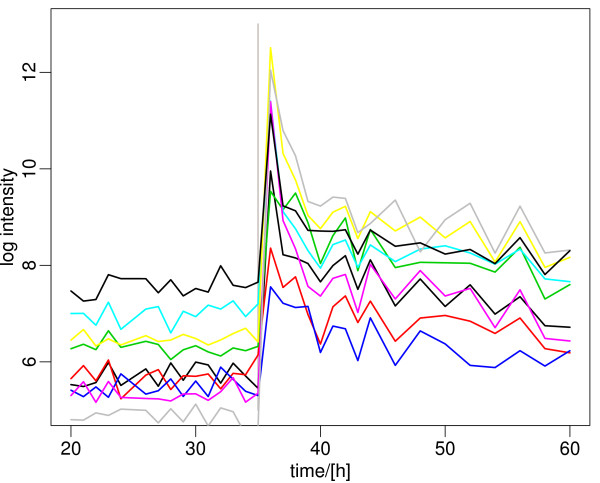
**Representative genes involved in synthesis of phosphate-free secondary polymers of the cell-wall that closely follow the expression profile of the phosphate transporters**. These genes are all part of a single genomic cluster (SCO4873-4882) and are part of the *pho *regulon. A list of the genes and their expression data is included in Additional File 2.

**Figure 8:**
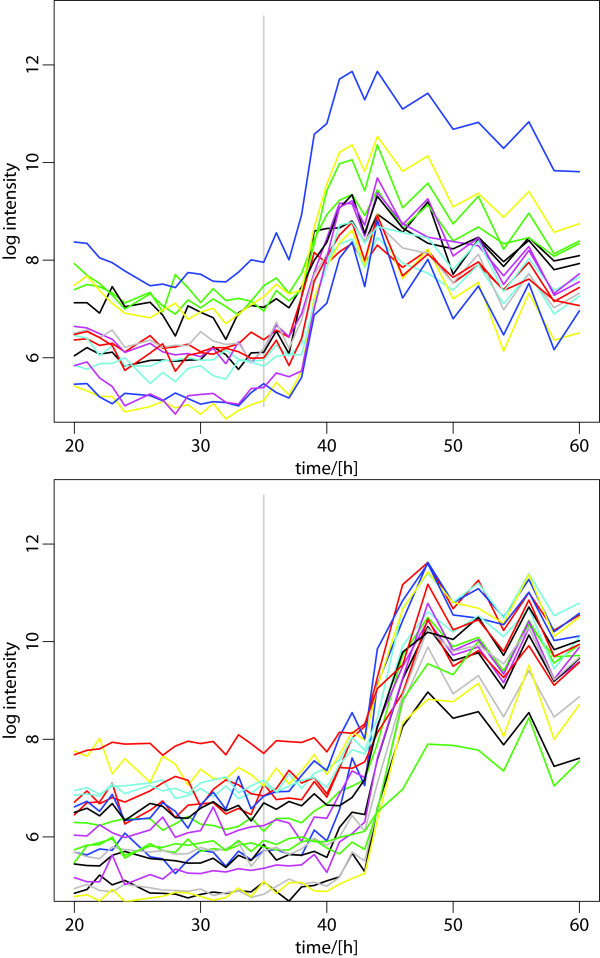
**Expression profile of the Red and Act antibiotics biosynthesis gene cluster**. The expression change of these genes, as a consequence of the metabolic switch, leads to the production of pigment antibiotics that characterizes the stationary growth phase of *Streptomyces*. Their upregulation is shifted by many hours, relative to the phosphate starvation, indicating a systematic delay in their regulatory mechanism. The gene expression levels are closely correlated to the increase in antibiotics levels measured in the medium. A list of the genes and their expression data is included in the supplementary table. Upper plot: Red cluster; lower plot: Act cluster.

The traditional metabolic switch is marked by a strong upregulation of the *pho *regulon, which include the phosphate regulator and transport genes (*phoR/P*, [19]; *pstSCAB*, [20]) and genes activated by *phoR/P *[20], which increase at 36 h, as soon as phosphate is depleted from the medium (Figure 6). Synchronously, the expression of a small gene cluster involved in the biosynthesis of a phosphate-free secondary polymer of the cell-wall increased [21] (SCO4876-4882, only excluding SCO4877; Figure 7). All of these 17 genes are among the 23 genes previously reported to be activated by PhoP [20-22]. This is followed by a gene expression change reflecting the classical signature of the metabolic switch in *Streptomyces*: first the gene cluster responsible for the biosynthesis of the pigmented antibiotic undecylprodigiosin (Red; [23]) is switched on at 38 h (Figure 8A), followed by the gene cluster responsible for the second pigmented antibiotic, actinorhodin (Act; [24]) after 43 h (Figure 8B). In both cases, several other genes along the genome show a strongly correlated expression pattern (see Additional File 2), identifying additional putative regulatory targets of the main transcription factors involved in enacting the metabolic switch, such as a small uncharacterized cluster of genes from SCO0392 to SCO0400.

## Discussion

**Figure 9:**
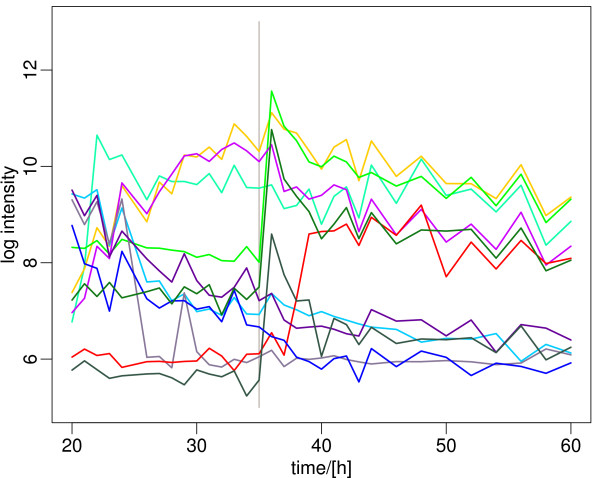
**Sequence of expression switches associated with the metabolic switch**. The expression profile of the ten most variable transcriptional regulators on the array is shown. As discussed in the main text, some of these regulators show an expression profile that clearly parallels that of the major expression clusters. These regulators are the main candidate drivers of the successive waves of gene expression that characterize the metabolic switch according to our study. They include phosphate transport system regulators (*phoP*, dark grey, *phoU*, light green, and *phoR*, dark green), the transcriptional regulator of the red cluster, *redD *(red), the pyrimidine regulatory protein *pyrR *(blue), as well as the nitrogen regulators *glnR *(light purple) and *glnK *(dark purple) [10]. A list of the genes and their expression data is included in Additional File 2.

Our data allow us to outline the complex series of expression switches associated with the metabolic switch in *Streptomyces*. To get a first idea of the regulatory events driving these successive waves of gene expression, we have focused our discussion on those major regulators that showed an interpretable gene expression profile, i.e. recognizable dynamics along the time course. Not all known regulators show such a clear expression pattern, and there are several reasons to explain this. For instance, posttranslational regulation can be more important for a particular regulator, or individual genes can hybridize less than optimal on the microarray, leading to expression signals that are too noisy for detailed interpretation. In such cases, additional orthogonal experimental approaches will be required. Figure 9 shows the most variable genes of this set. It is obvious that many of them closely parallel the expression profile of some of the major clusters: the phosphate transport system regulator SCO4228/PhoU [20] and the phosphate starvation response regulator SCO4229/PhoR and SCO4230/PhoP [19] peak at the onset of phosphate depletion. The expression of *phoU *is activated by PhoP [20], consistent with the observed expression pattern. The transcriptional regulator SCO5877/RedD [25] switch on about 3 h after the main metabolic switch, when the Red cluster of antibiotics biosynthesis genes is induced. Several less well characterized putative regulators closely follow the expression pattern of the developmental gene cluster that is repressed upon the metabolic switch. Determining the mechanism of regulation using statistical causal inference in combination with targeted gene disruption experiments will be the next important step in understanding this complex system. In addition, flux balance analysis will be useful for correlating the observed sequence of expression changes to predicted re-arrangements of metabolic fluxes in central metabolism during the metabolic switch. It will be particularly important to examine other limitation conditions (e.g., carbon or nitrogen depletion) for differences and similarities in their metabolic switch.

## Conclusions

In addition to the genes discussed here, most of which have been studied in detail before, there is a large number of unannotated genes showing the same strong expression switches. For all genes, the complete expression profiles have been deposited in GEO for further exploration (http://www.ncbi.nlm.nih.gov/geo/: GSE18489, GPL9417, GSM460281-460312).

It is evident from our data that gene expression during the metabolic switch is far more dynamic than initially expected. Our densely sampled time-series allows identification of rapid complex expression changes of biological significance, as well the prediction of central regulatory relationships.

## Methods

### Bacterial strain and cultivation conditions

Experiments were performed using *S. coelicolor *A3(2) strain M145 [26]. Cultivations were performed in 3-liter fermentors (Applikon) with an initial culture volume of 1.8 liter. The growth medium used was based on ion-free water and consisted of Na-glutamate, 55.2 g/l; glucose, 40 g/l; MgSO4, 2.0 mM; phosphate, 4.6 mM; supplemented minimal medium trace element solution [15], 8 ml/l and TMS1, 5.6 ml/l. TMS1 consisted of FeSO_4 _× 7 H_2_O, 5 g/l; CuSO_4 _× 5 H_2_O, 390 mg/l; ZnSO_4 _× 7 H_2_O, 440 mg/l; MnSO_4 _× H_2_O, 150 mg/l; Na_2_MoO_4 _× 2 H_2_O, 10 mg/l; CoCl_2 _× 6 H_2_O, 20 mg/l, and HCl, 50 ml/l. Furthermore, the pH was adjusted to 7.0 by addition of 2 M NaOH and 1.8 ml Clerol FBA 622 fermentation defoamer (Diamond Shamrock Scandinavia) were added to the growth medium before inoculation, while additional 0.5 ml were added after 58 hours. For the inoculum, 10^9 ^CFU of *S. coelicolor *spores (typically 1 ml of a thawed spore-stock in 20% (v/v) glycerol) were germinated for 5 hours at 30°C and 250 rpm in 250 ml baffled shake-flasks with 2 grams of 3 mm glass beads containing 50 ml 2× YT medium [15]. The germinated spores were harvested by centrifugation (3200 × g, 15°C, 5 min) and re-suspended in 5 ml ion-free water. Each fermentor (1.8 liter growth medium) was inoculated with 4.5 ml germinated spores suspension. Throughout the fermentation trial, pH 7.0 was maintained by automatic addition of 2 M HCl (typically 150 ml per fermentor). Dissolved oxygen was maintained at a minimum of 50% by automatic adjustment of the agitation speed and a constant aeration rate of 0.9 l/min air. The agitation speed range was from approximately 300 rpm (set minimum) to 1050 rpm. Dissolved oxygen, agitation speed and CO_2 _evolution was measured and logged online, while samples for the determination of cell dry weight, levels of growth medium components and secondary metabolites were taken throughout the fermentation trial. Samples for transcriptome analysis were taken hourly from 20 to 44 hours, and every two hours from 44 to 60 hours: 3 × 4 ml culture sample were applied in parallel onto three 0.45 μm nitrocellulose filters (Millipore) connected to vacuum. The biomass on each filter was immediately washed twice with 4 ml double-autoclaved ion-free water pre-heated to 30°C, before the filters were collected in a 50 ml plastic tube, frozen in liquid nitrogen and stored at -80°C until RNA isolation. The net culture volume reduction over the time-course of the fermentation was typically 930 ml.

### Analyses of nutrient levels and secondary metabolites

Levels of phosphate, glucose and glutamate were measured spectrophotometrically by using the Spectroquant Phosphate test kit, the Lactose/D-glucose test kit (both R-Biopharm), and the L-glutamate Bioassay kit (USBiological), respectively, following the manufacturer's instructions. Extracellular actinorhodin (γ-actinorhodin) levels were determined spectrophotometrically at 608 nm after adjusting the supernatants to pH 12 with NaOH; undecylprodigiosin levels were determined spectrophotometrically at 530 nm after acidified methanol extraction from the mycelium [6]. Total blue pigments levels were determined by extraction of whole culture samples with 1 M KOH (final concentration) and spectrophotometric measurement at 640 nm [27].

### RNA isolation and transcriptome analysis

RNA was isolated from each sample using phenol extraction and the RNeasy Midi Kit (Qiagen). Affymetrix genechip arrays were designed by Affymetrix with 13 25 bp-oligos targeting each coding sequence. The array contained a total of 226,576 perfect match probes (oligos of length 25 bp), including 8205 probe sets targetting coding sequences, 10834 intergenic probe sets (sense and antisense), and 3671 probe sets targetting predicted non-coding RNAs. Biotinylated cDNA was prepared after fragmentation according to the standard Affymetrix protocol from 3 μg total RNA. 3 μg of cDNA were hybridized for 16 hr at 50°C and arrays were scanned using the Affymetrix GeneChip Scanner 3000 7G. Gene expression values were normalized using RMA [28] and clustered based on pair-wise Pearson correlation. Details on the array layout and R scripts [29] used for the analysis are available from the authors. All expression data have been deposited in GEO (accession numbers GSE18489, GPL9417, GSM460281-460312). In-depth analysis was performed within the visual analysis framework Mayday [30].

### qRT-PCR validation of expression

qRT-PCR was performed in 384-well format with the LightCycler 480 System (Roche) and the QuantiTect SYBR Green PCR Kit (Qiagen). The principal sigma-like transcriptional factor of *S. coelicolor *(hrdB) was used as reference (housekeeping) gene.

## Authors' contributions

All authors are members of the SysMO-STREAM consortium and contributed to the design and execution of the study, and the discussion of the results. EMHW coordinated the study. PB, AW, OMJ, HS and TEE performed the fermentations and provided samples for transcriptomics. KN designed the microarrays. KN, FB, AH and MB performed the microarray and qRT-PCR analysis. KN, FB, AH, JM, ERM, MJH, RL, DAR, DLW, NJB and RB performed bioinformatics analyses. ARG, JR, WW, MCMS, JFM, DAH and ET contributed to the biological interpretation. RB and ET drafted the manuscript. All authors read and approved the final manuscript.

## Supplementary Material

Additional file 1 **Validation of the expression data by qRT-PCR**. Black lines show the array data, red lines indicate the corresponding PCR measurements. Click here for file

Additional file 2 **Description of individual genes in the clusters shown in **Figures 2, 3, 4, 5, 6, 7, 8, 9. The columns specify the SCO gene identifier, the gene name (if available), a brief description of the gene's function and the normalized expression value of the corresponding microarray probe set for each time point. Click here for file
