# Supplementary figures and images for: The dynamic architecture of the metabolic switch in Streptomyces coelicolor
Source: BMC Genomics. 2010 Jan 6;11:10. doi: 10.1186/1471-2164-11-10 (PMC2824715; doi:10.1186/1471-2164-11-10)

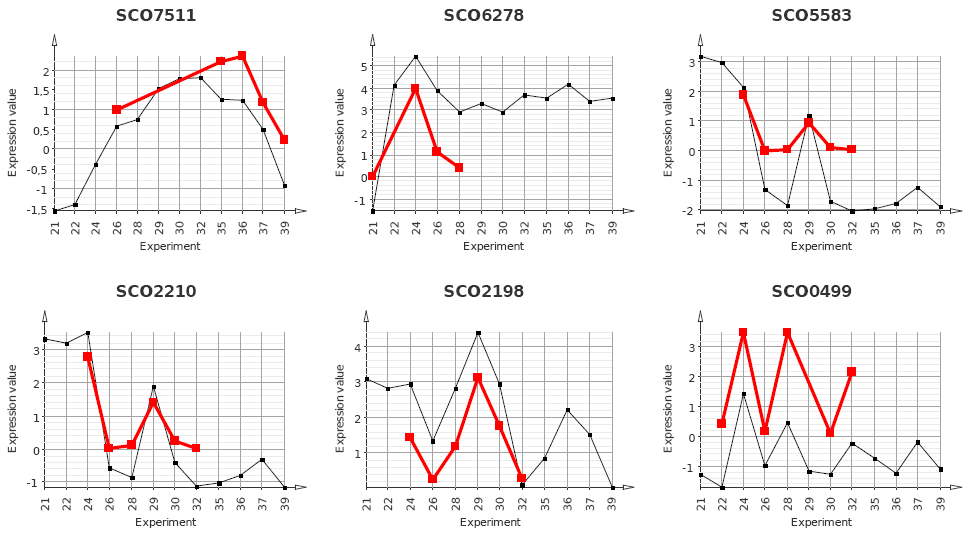

Supplement: Additional file 1 — Validation of the expression data by qRT-PCR. Black lines show the array data, red lines indicate the corresponding PCR measurements. [file 1471-2164-11-10-S1.PNG]
